# Prevalence and Predictors of Domestic Violence in India: Complex Sample Analysis of a Nationally Representative Study Conducted Between 2019 and 2021

**DOI:** 10.7759/cureus.66113

**Published:** 2024-08-04

**Authors:** Sanjeeb K Mishra, Gourahari Pradhan, Subrat K Pradhan, Gitarani Choubey

**Affiliations:** 1 Field Epidemiology Training Program, Indian Council of Medical Research, Chennai, IND; 2 Community Medicine, Veer Surendra Sai Institute of Medical Sciences and Research, Sambalpur, IND; 3 Pulmonary Medicine, Veer Surendra Sai Institute of Medical Sciences and Research, Sambalpur, IND; 4 Forensic Medicine, Veer Surendra Sai Institute of Medical Sciences and Research, Sambalpur, IND

**Keywords:** nfhs-5, regression analysis, demographic and health survey, spouse abuse, domestic violence, sustainable development goal

## Abstract

Background: Violence against women has been one of the dreaded social evils that humanity is facing. There have been concerted efforts to eliminate this evil, and sustainable development goals goal 5.2.1 gave it a timeline. The current study was carried out to estimate the burden of domestic violence (DV) against women and to investigate the sociodemographic correlates of DV victims in India.

Methods: Data were drawn from the fifth National Family Health Survey round. According to Demographic Health Survey guidelines, DV is measured using a 13-item questionnaire in the women's survey. Complex sample analysis was done using a primary sampling unit, sample weight, and stratification variables to estimate the weighted prevalence. Chi-square and multivariate logistic regression determine the unadjusted and adjusted odds ratio. The analysis is carried out using SPSS version 26 (IBM Corp., Armonk, NY).

Results: The weighted prevalence of DV against women in India in 2019-2021 was 31.2%. Approximately 28.5%, 13.1%, and 5.7% of women reported experiences of physical, emotional, and sexual violence, respectively. Karnataka was the worst affected state, with 47.3% of women facing DV. Individual factors like education and occupation, household factors like husband's education, occupation, drinking habit, wealth index, and community-level factors like caste, religion, and place of residence were significant predictors of DV. Lower levels of education and lower socioeconomic status were essential predictors of DV.

Conclusion: The importance of education for both females and males has repeatedly been directly associated with DV, but the interventions have failed to improve the situation and warrant a new strategy. Awareness about the legal consequences of DV in lower socioeconomic classes also has the potential to cut down the numbers. Further research into the causality can improve the planning for better intervention modalities.

## Introduction

Gender-based violence (GBV) against women is a pressing global issue affecting one in every three women throughout their lives. The sustainable development goals emphasize the crucial need to address gender bias and eliminate violence against women and girls in all its forms [1]. GBV against women is defined as “any action causing physical, sexual, or mental harm to women, including threats, coercion, or unjust deprivation of liberty, whether in public or private settings” [2]. World Health Organization (WHO) identifies it as a significant public health issue and a violation of women's human rights. Their estimates indicate that one in three women worldwide experiences GBV, with most incidents involving interpersonal violence [3]. This violence has severe short- and long-term consequences for women's physical, mental, sexual, and reproductive health, as well as affects the well-being of their children. Additionally, it results in substantial social and economic costs, ranging from mental health issues like depression and post-traumatic stress to physical harm and even death [4].

Taboo, bias, lack of free speech, and improper reporting have been postulated to have underreported the prevalence of violence against women, giving rise to the “Iceberg” of domestic violence (DV) [5]. The WHO has reported the Southeast Asian region to have the highest burden of violence against women. Violence against women remains unreported in many parts of India daily [6]. According to the National Crime Records Bureau (2021), the crime rate per lakh female population stood at 64.5 in 2021, up from 56.5 in 2020. This amounts to a crime against women every minute in India [7]. The fifth National Family Health Survey (NFHS)-4 report indicated that 33% of ever-married women had experienced physical, sexual, or emotional spousal violence [8-10]. This forms the background for our analysis. The NFHS-5 round was conducted from 2019 to 2021. The DV questionnaire was a key instrument in collecting the information. We analyzed the data contained in this nationally representative study to estimate the prevalence and determine the predictors of DV among women in India.

## Materials and methods

Data source

We used the data from NFHS-5 conducted in 2019-2021 for analysis in this study. The NFHS-5 is a nationally representative survey conducted across India's states and union territories. It gathered information from 636,699 households, 724,115 women, and 101,839 men, covering all the 707 districts in the country [11].

NFHS-5 used a stratified sample design, dividing districts into urban and rural areas. Rural areas were stratified by village size, creating three explicit strata and six equal-sized substrata. Villages were selected using probability proportional to size (PPS) sampling from the 2011 census sampling frame. In each district, the sample was chosen in two stages: the first stage involved selecting primary sampling units (PSUs), which encompassed villages in rural areas and census enumeration blocks in urban areas, using PPS. The second stage randomly selected an equal number of houses within each PSU. Systematic sampling was used to select the households. In each state, the number of families chosen per PSU was 20. It included 30,456 PSUs, with 7,910 urban and 22,546 rural. A detailed description of the sampling design and survey procedure is provided in the NFHS-5 national report [12]. The NFHS-5 provided information for 724,115 women, out of whom 494,830 were selected for interviews on DV. However, 72,320 women could only be interviewed for whom information about violence was available. Among them, 8,469 were never in any union and were filtered out. The remaining 63,851 married, divorced, separated, or widowed records have been considered for the prevalence and predictor analysis.

Outcome variables

The present study considered DV as the dependent variable. The violence was measured in NFHS-5 by asking all ever-married women if their husbands ever committed the following to them: (1) shook, pushed, or threw something at her, (2) slapped her, (3) twisted her arm or pulled her hair, (4) punched her with his fist, (5) kicked, dragged, or beaten her up, (6) tried to choke or burn her on purpose, or (7) threatened or attacked with a knife, gun, or any other weapon as components of physical violence. Sexual violence includes whether the woman was (1) physically forced to have sexual intercourse, (2) physically forced to perform any other sexual acts, or (3) forced with threats or in any other way to perform sexual acts when she did not want to. Similarly, emotional violence against women by the husband was judged by three questions: (1) humiliated in front of others, (2) threatened to hurt or harm, or (3) insulted or made her feel bad about herself. With this information, binary variables were created for any physical violence, sexual violence, or emotional violence. The final variable, i.e., DV, was developed with three responses: (1) experience of any physical violence, (2) experience of any sexual violence, and (3) experience of any emotional violence.

Covariates

This study examined additional factors likely to be associated with DV. Demographics include the woman’s age. Women's educational attainment was categorized as no education, primary, secondary, and higher education. The husband's educational status was also considered. Caste, religion, domicile, and household wealth were included as socioeconomic variables. Caste was considered because it has been a basis of discrimination for a long time, and the government has taken affirmative action, including reservation, to bring about social equality. Castes were grouped into scheduled caste (SC), scheduled tribe (ST), other backward classes (OBC), and others (i.e., general). In assessing domestic abuse, religious belonging is also considered a confounding element. Hinduism, Islam, and other religions were classified. The type of habitation was included to investigate the differences between rural and urban areas. The household wealth index was calculated utilizing the ownership of household assets, including homes. We utilized the framework used by Haobijam and Singh, modified as per our variables for the analysis [13].

Statistical analyses

Univariate statistics adjusted for sample weight and the PSU were used to describe the distribution of key predictors, confounders, and outcome variables. The regression results were presented as crude and adjusted odds ratios (AORs) with a 95% confidence interval (CI). All the statistical analyses were performed using SPSS version 26.0 (IBM Corp., Armonk, NY).

## Results

Our analysis included 63,851 numbers of women (15-49 years) in India in 2020. The mean age of the participants was 33.9 (SD, 8 years); 48,363 (75.7%) were from rural areas, and 60,480 (94.7%) were currently married. Regarding educational status, 57,028 (89.3%) of the women had education up to secondary level, and only 23,236 (36.4%) were working women. Three-fourths of women were Hindus, with a predominance of OBC (38.6%), and mostly belonged to a family headed by a male. Most of the women, 58,736 (92%), had children, and only 3,033 (4.8%) of women were pregnant at the time of the survey.

The mean age of the participants' husbands was 38 years, with an SD of nine years. The distribution of the educational status of husbands was similar to that of women, with 54,798 (86.2%) educated up to the secondary level. Agriculture was the main occupation for 23,834 (37.3%) of husbands, followed by skilled/unskilled workers at 28.9% (18,428). Sociodemographic characteristics are detailed in Table 1.

**Table 1:** Characteristics of study participants (women in DV module) of age 15-49 years in India (N = 63,851) SC: scheduled caste; ST: scheduled tribe; OBC: other backward classes; DV: domestic violence

Variables	Categories	n (%)
Age	≤30	20,814 (32.6)
	31-40	25,316 (39.7)
	≥41	17,721 (27.8)
Place of residence	Urban	15,488 (24.3)
	Rural	48,363 (75.7)
Marital status	Married	60,480 (94.7)
	Widowed/divorced/separated	3,371 (6.3)
Wealth index	Poorest	13,222 (20.7)
	Poorer	13,566 (21.2)
	Middle	12,977 (20.3)
	Richer	12,383 (19.4)
	Richest	11,703 (18.3)
Education	No education	18,783 (29.4)
	Primary	9,302 (14.6)
	Secondary	28,943 (45.3)
	More than secondary	6,823 (10.7)
Occupation	Not working	40,615 (63.6)
	Working	23,236 (36.4)
Husband's age	≤30	13,530 (21.2)
	31-40	23,673 (37.1)
	≥41	23,277 (36.5)
	Missing	3,371 (5.3)
Husband's education	No education	11,628 (18.2)
	Primary	9,268 (14.5)
	Secondary	33,902 (53.1)
	More than secondary	8,819 (13.8)
Husband's occupation	Professional	4,735 (7.4)
	Clerk/sales/services	13,430 (21.1)
	Agricultural	23,834 (37.3)
	Skilled/unskilled manual	18,428 (28.9)
	Other	3,424 (5.4)
Religion	Hindu	48,548 (76.0)
	Muslim	7,585 (11.9)
	Christian	4,570 (7.2)
	Others	3,148 (4.8)
Caste	SC	12,164 (19.1)
	ST	12,220 (19.1)
	OBC	24,661 (38.6)
	Others	11,717 (18.4)
	Missing	3,089 (4.8)
Current pregnancy status	Yes	3,033 (4.8)
	No	60,818 (95.2)
Having live children	Yes	58,736 (92.0)
	No	5,115 (8.0)
Gender of the family head	Male	53,581 (83.9)
	Female	10,270 (16.1)

**Figure 1:**
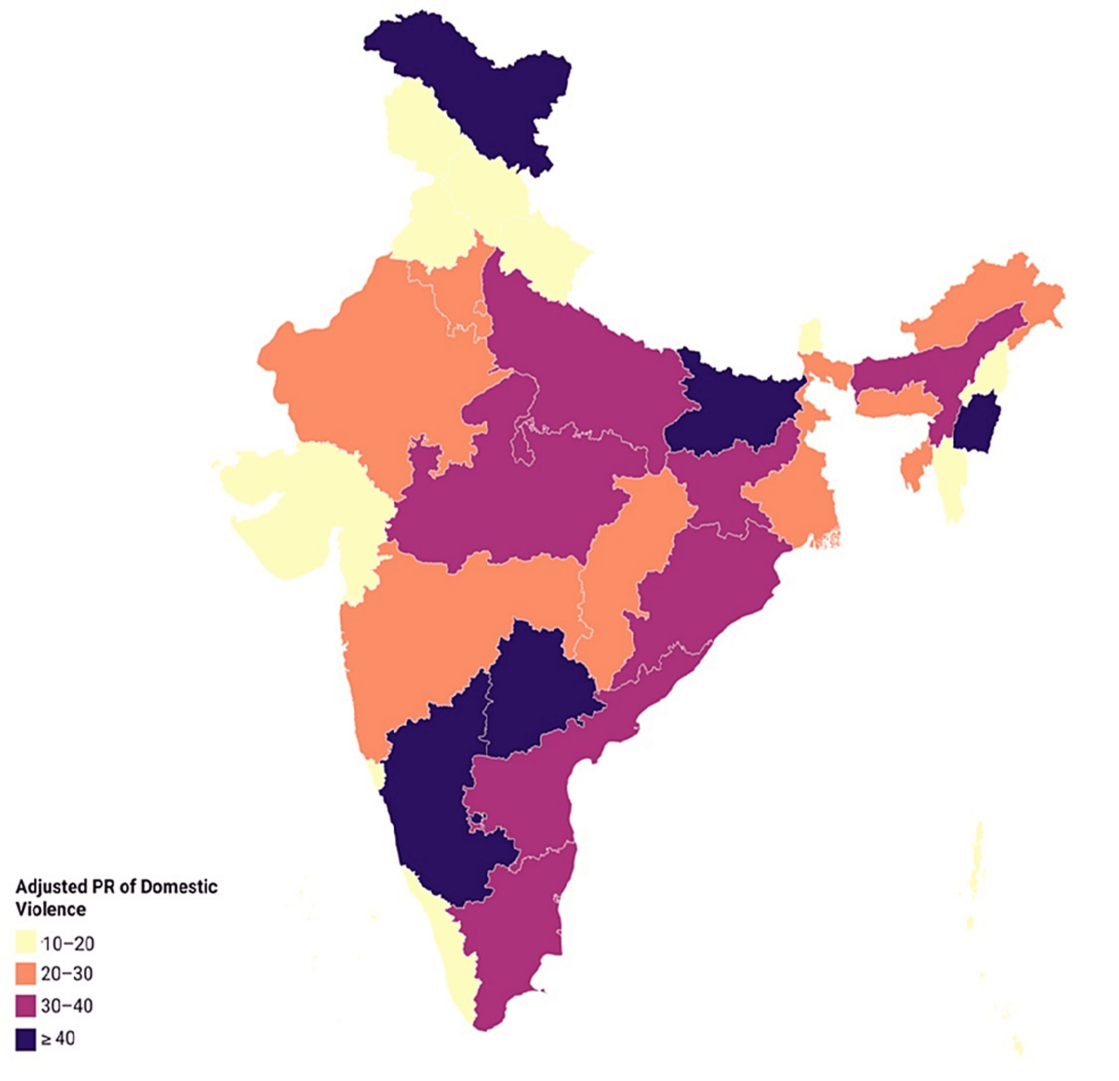
Statewise weighted prevalence of DV among women aged 15-49 years (N = 63,851) in 2019-2021 PR: prevalence ratio; DV: domestic violence

The weighted prevalence of DV was estimated to be 31.2%. Approximately 28.5%, 13.1%, and 5.7% of women reported experiences of physical, emotional, and sexual violence, respectively. Among these, 2,110 (3.1%) women were exposed to all forms of DV. Severe physical violence was found among 8.4% of the interviewed women. Karnataka (47.3%), Bihar (42.0%), Manipur (40.4%), Ladakh (40.2%), and Telangana (40.2%) were among the states with the highest prevalence of DV, whereas Lakshadweep (0.8%) and Goa (9.8%) had the lowest prevalence (Figure 1).

**Figure 2:**
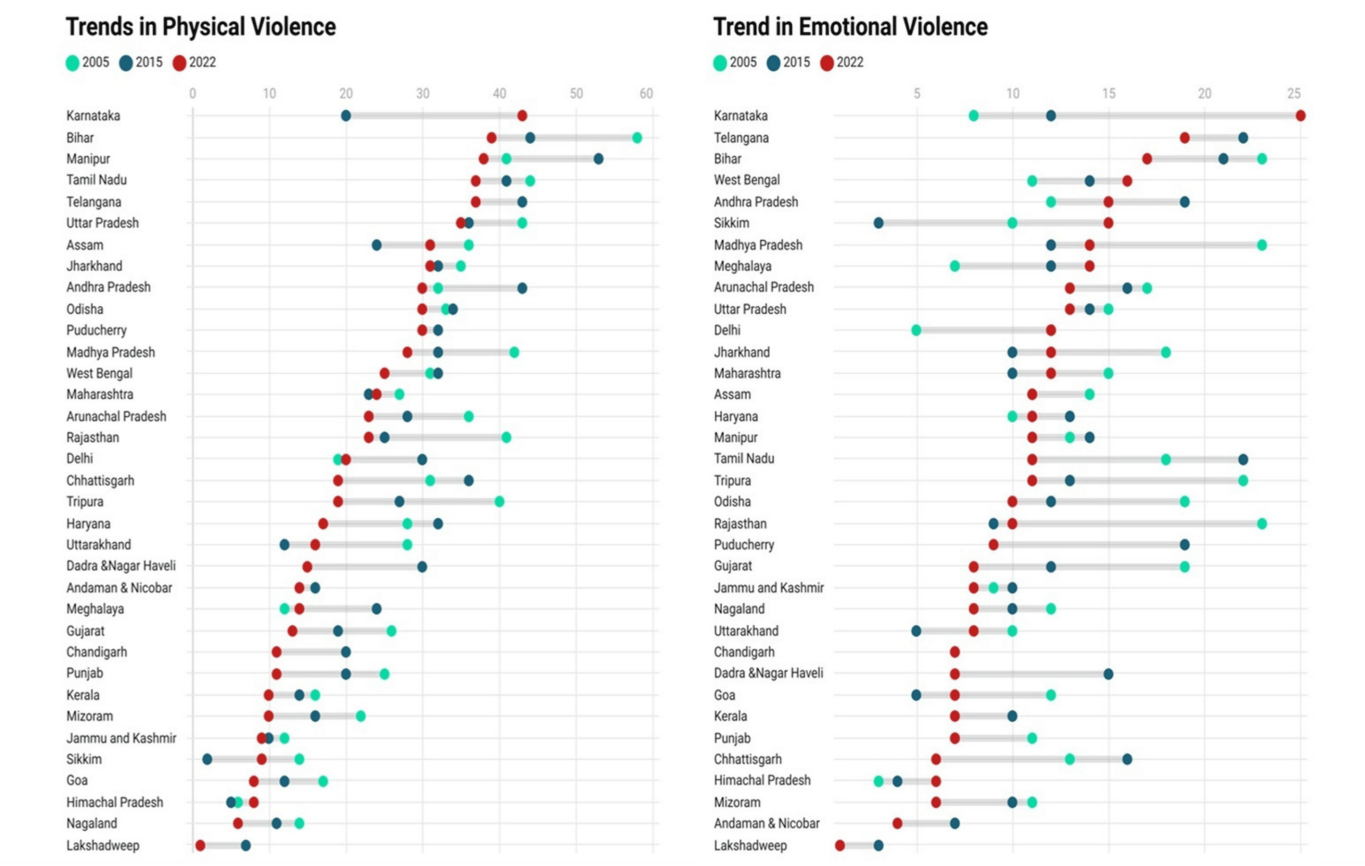
Trend in physical and emotional violence from NFHS-3 to NFHS-5 NFHS: National Family Health Survey

Karnataka (43.1%) topped in physical violence, followed by Bihar (39.4%) and Manipur (38%) (Figure 2). Karnataka (24.7%) again topped in emotional violence, followed by Ladakh (18.7%) and Telangana (18.5%) (Figure 2).

**Figure 3:**
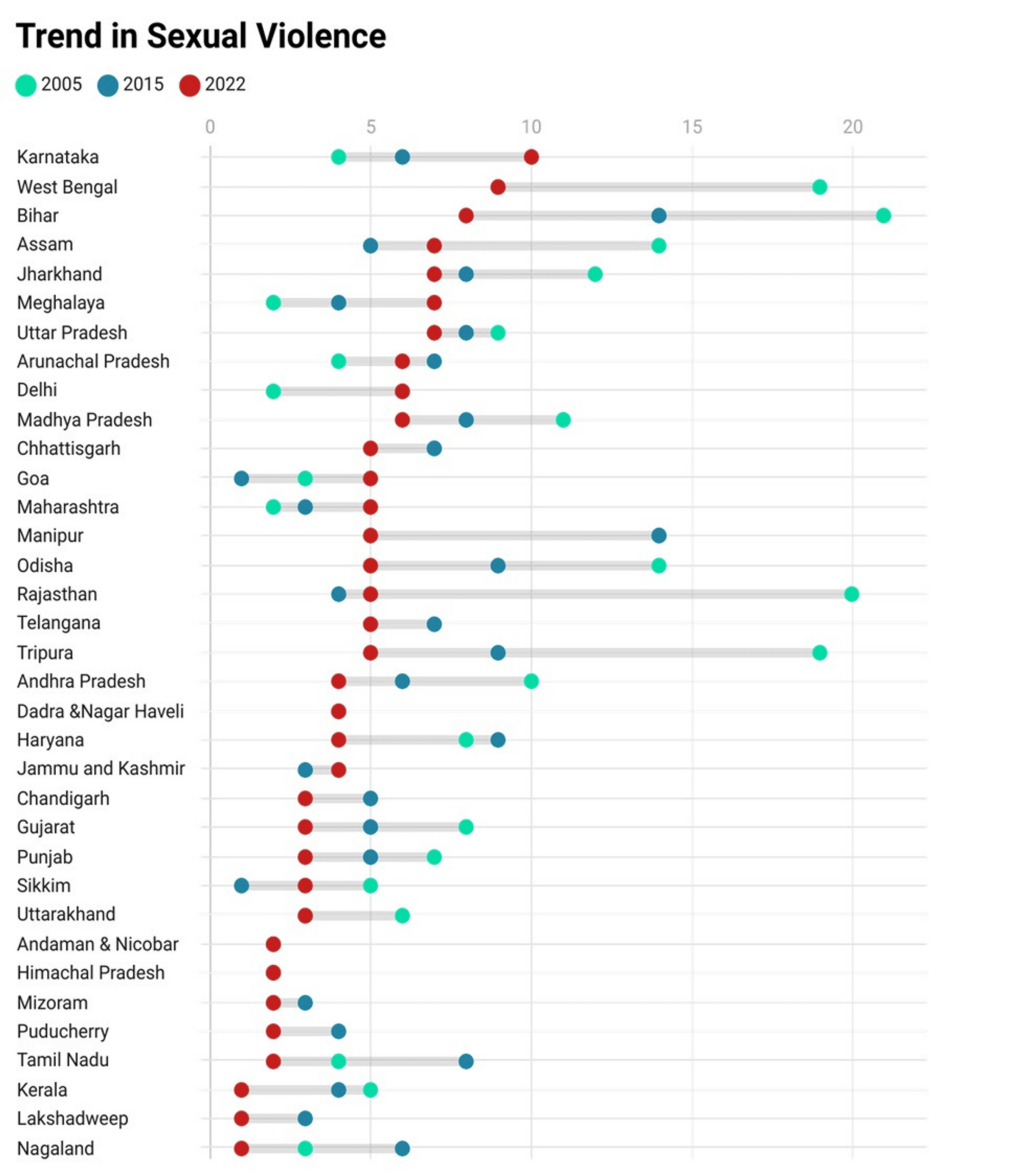
Trend in sexual violence from NFHS-3 to NFHS-5 NFHS: National Family Health Survey

Concerning sexual violence, West Bengal (8.7%) and Bihar (8.1%) followed Karnataka at the top (9.8%) (Figure 3).

Chi-square test of independence

A chi-square test of independence was performed to assess the relationship between the DV and descriptive variables. The results indicated that DV was significantly associated with all the descriptive variables. Details of the chi-square test results are shown in Table 2.

**Table 2:** Chi-square test of independence between sociodemographic characteristics with DV among women aged 15-49 years in India (N = 63,851) PR: prevalence ratio; CI: confidence interval; SC: scheduled caste; ST: scheduled tribe; OBC: other backward classes; DV: domestic violence

Variables	Categories	DV, n (%)		Weighted PR (95% CI)	χ^2^	p value
		Yes	No			
Age	≤30	6,807 (29.4)	17,549 (70.6)	0.88 (0.85-0.92)	53.5	0.001
	31-40	7,477 (32.1)	16,996 (67.9)	0.97 (0.93-1.01)		
	≥41	4,751 (33.1)	10,271 (66.9)	Reference		
Education	No education	7,062 (39.1)	11,721 (60.9)	2.24 (2.05-2.47)	1,407.1	<0.001
	Primary	3,098 (35.7)	6,204 (64.3)	2.05 (1.87-2.26)		
	Secondary	7,744 (28.7)	21,199 (71.3)	1.65 (1.50-1.81)		
	More than secondary	1,131 (17.4)	5,692 (82.6)	Reference		
Occupation	Not working	10,641 (27.8)	29,974 (72.2)	0.73 (0.70-0.76)	562.8	<0.001
	Working	8,394 (37.9)	14,842 (62.1)			
Husband’s age	≤30	3,540 (27.4)	9,990 (72.6)	0.86 (0.82-0.90)	93.5	<0.001
	31-40	6,976 (31.1)	16,697 (68.9)	0.98 (0.94-1.02)		
	≥41	7,169 (31.9)	16,108 (68.1)	Reference		
Husband's education	No education	4,557 (41.1)	7,071 (58.9)	2.14 (1.99-2.30)	1,252.3	<0.001
	Primary	3,298 (37.5)	5,970 (62.5)	1.95 (1.81-2.10)		
	Secondary	9,446 (29.6)	24,456 (70.4)	1.54 (1.44-1.65)		
	More than secondary	1,657 (19.2)	7,162 (80.8)	Reference		
Husband's occupation	Professional	903 (20.9)	3,832 (79.1)	0.58 (0.42-0.65)	656.7	<0.001
	Clerk/sales/services	3,362 (27)	10,068 (73)	0.76 (0.72-0.80)		
	Skilled/unskilled manual	5,890 (33.1)	12,538 (66.9)	0.93 (0.89-0.97)		
	Other	902 (28.5)	2,522 (71.5)	0.8 (0.73-0.86)		
	Agricultural	7,978 (35.5)	15,856 (64.5)	Reference		
Wealth index	Poorest	5,012 (40.2)	8,210 (59.8)	1.98 (1.85-2.13)	981.5	<0.001
	Poorer	4,460 (35.2)	9,106 (64.8)	1.73 (1.62-1.86)		
	Middle	3,939 (31.6)	9,038 (68.4)	1.56 (1.45-1.67)		
	Richer	3,339 (28.2)	9,044 (71.8)	1.39 (1.30-1.49)		
	Richest	2,285 (20.3)	9,418 (79.7)	Reference		
Marital status	Married	17,685 (30.6)	42,795 (69.4)	0.7 (0.66-0.75)	188	<0.001
	Widowed/divorced/separated	1,350 (43.4)	2,021 (56.6)			
Husband drinks alcohol	Yes	8,306 (51.4)	9,526 (48.6)	2.1 (2.01-2.16)	2,758.1	<0.001
	No	10,729 (24.6)	35,290 (75.4)			
Having live children	No	1,162 (21.7)	3,953 (78.3)	0.72 (0.65-0.8)	163.1	<0.001
	Yes	17,873 (30)	40,863 (70)			
Current pregnancy status	Yes	750 (25.4)	2,283 (74.6)	0.83 (0.74-0.87)	30.7	0.007
	No	18,285 (31.6)	42,533 (68.4)			
Gender of the family head	Male	15,814 (30.8)	37,767 (69.2)	0.9 (0.87-0.95)	16.9	0.031
	Female	3,221 (33.9)	7,049 (66.1)			
Place of residence	Urban	4,035 (27.2)	11,453 (72.8)	0.82 (0.77-0.87)	192.9	<0.001
	Rural	15,000 (33.1)	33,363 (66.9)			
Religion	Hindu	15,218 (32)	33,330 (68)	1.4 (1.27-1.54)	364.3	<0.001
	Muslim	2,199 (30.2)	5,386 (69.8)	1.33 (1.18-1.49)		
	Others	1,618 (22.8)	6,100 (77.3)	Reference		
Caste	SC	4,219 (36.4)	7,945 (63.6)	1.51 (1.41-1.61)	127.3	<0.001
	ST	3,478 (33.8)	8,742 (66.2)	1.4 (1.30-1.51)		
	OBC	7,883 (32.2)	16,778 (67.8)	1.33 (1.26-1.41)		
	Others	2,659 (24.1)	9,058 (75.9)	Reference		

Regression analysis

McFadden pseudo-R^2^ suggests that the predictors in the complex sample logistic regression model can explain 7.1% of the variance in outcome variables. Sociodemographic factors such as residence, occupation, education, caste, religion, wealth index, having live children, and the husband's drinking habits were significantly associated with DV. Details of the odds ratio with its corresponding 95% CIs are shown in Table 3.

**Table 3:** Complex sample regression analysis between sociodemographic characteristics with DV among women aged 15-49 years in India (N = 63,851) AOR: adjusted odds ratio; CI: confidence interval; SE: standard error; SC: scheduled caste; ST: scheduled tribe; OBC: other backward classes; DV: domestic violence

Variables	Categories	AOR (95% CI)	p value	SE
Age	≤30	1.00 (0.86-1.18)	0.978	0.08
	31-40	1.01 (0.9-1.14)		0.06
	≥41	Reference		-
Education	No education	1.71 (1.44-2.04)	0.0001	0.09
	Primary	1.44 (1.20-1.71)		0.09
	Secondary	1.28 (1.10-1.49)		0.08
	More than secondary	Reference		-
Occupation	Not working	0.76 (0.70-0.83)	0.0001	0.04
	Working	Reference		-
Husband’s age	≤30	0.99 (0.84-1.17)	0.292	0.08
	31-40	1.07 (0.95-1.2)		0.06
	≥41	Reference		-
Husband's education	No education	1.27 (1.07-1.50)	0.033	0.09
	Primary	1.22 (1.03-1.44)		0.09
	Secondary	1.12 (0.98-1.29)		0.07
	More than secondary	Reference		-
Husband's occupation	Professional	0.78 (0.66-0.93)	0.008	0.09
	Clerk/sales/services	0.85 (0.76-0.94)		0.06
	Skilled/unskilled manual	0.91 (0.82-1.01)		0.05
	Other	0.8 (0.67-0.97)		0.09
	Agricultural	Reference		-
Wealth index	Poorest	1.66 (1.41-1.93)	0.0001	0.08
	Poorer	1.52 (1.32-1.75)		0.07
	Middle	1.3 (1.15-1.48)		0.07
	Richer	1.27 (1.12-1.44)		0.07
	Richest	Reference		-
Marital status	Married	0.7 (0.66-0.75)	0.0001	0.05
	Widowed/divorced/separated	Reference		-
Having live children	No	0.69 (0.58-0.80)	0.0001	0.08
	Yes	Reference		-
Current pregnancy status	Yes	1.05 (0.87-1.26)	0.629	0.09
	No	Reference		-
Gender of the family head	Male	1.03 (0.92-1.15)	0.648	0.06
	Female	Reference		-
Husband drinks alcohol	Yes	2.1 (2.56-3.03)	0.0001	0.04
	No	Reference		-
Place of residence	Urban	0.88 (0.78-0.99)	0.042	0.06
	Rural	Reference		-
Religion	Hindu	1.75 (1.52-2.02)	0.0001	0.08
	Muslim	2.51 (2.11-2.98)		0.10
	Others	Reference		-
Caste	SC	1.14 (0.99-1.31)	0.0001	0.07
	ST	0.82 (0.69-0.97)		0.09
	OBC	1.02 (0.89-1.16)		0.07
	Others	Reference		-

## Discussion

The present study delves into the prevalence and factors associated with DV based on a comprehensive analysis of data from 63,851 ever-married women across India. It is essential to scrutinize the findings through a lens of diversity, recognizing the multifaceted nature of the sample and the intricate sociocultural landscape of India. The prevalence of DV has steadily declined from NFHS-2 (52%), NFHS-3 (37%), and NFHS-4 (33.3%) to 31% in this analysis but remains high, affecting one in every third of women [9,10].

One striking observation is the significant regional variation in the prevalence of DV. While the overall prevalence has declined nationally, Karnataka stands out with a concerning rise compared to 2005. The rise in figures in Karnataka is difficult to explain, but the lockdown can be one of the possible explanations for two reasons. First, women's employment in Karnataka is higher at 45% compared to 36% at the national level, and they were forced to remain indoors due to the lockdown. Second, the lockdown did lead to financial stress in almost all families, which could have fueled DV. Another key point that could have contributed to the numbers is that empowered women report their experiences without hesitation compared to dependent women [5]. On the contrary, Manipur has shown a 15% decline from the NFHS-4 level. Low prevalence in Goa and Kerala can be attributed to high literacy rates to be aware of the consequences of possible imprisonment for DV [14,15]. Bihar has also been able to bring a substantial decline in all forms of violence compared to 2005 and 2015 levels (more than 20% reduction). Bihar's notable reduction in violence, attributed to alcohol prohibition, emphasizes the role of policy interventions in shaping DV dynamics [16,17]. These regional variations highlight the need for targeted, region-specific strategies to address DV effectively.

Personal factors like education and occupation are significant at a 5% level. The risk of DV decreases with an increase in the education level of women and their husbands. This is self-explanatory: education of husbands leads to respect for women and knowledge of the legal complications of domestic abuse. The increase in women’s education has contributed to the improvement in conflict management skills, thus playing a pivotal role in mitigating challenging situations. Findings from NFHS-4, as well as other studies, also corroborate the current findings [8,18-21]; occupational factors also play a crucial role, with working women facing a higher risk of DV. The empowerment associated with employment may challenge traditional patriarchal norms, potentially contributing to increased vulnerability. Similar results were explained in a study conducted on NFHS-4 data [8,13,21]. Women whose husbands were in agricultural occupations had the highest odds for DV, followed by skilled/unskilled manual workers. Professional husbands were less likely to resort to DV. This is also in line with other studies and can be explained by the education gradient across these occupations. The odds of experiencing DV decrease as we move up the income ladder, as economic deprivation can lead to family stress and strain that contribute to DV. Similar results were explained by studies across the globe, highlighting the positive influence of economic stability on family health [13,14,19,22,23].

Current pregnancy was not significantly associated with DV, which is similar to findings from other studies in India [8,13] and nearby areas [24]. However, this is in contrast to a study reported from Portugal, which reported a higher prevalence of DV during pregnancy [25]. Having live children increased the odds of DV by 1.44 times, and the association was statistically significant and is in line with the findings of a systematic review on DV [26]. This finding needs further exploration into the gender of the child as women are under pressure to deliver male babies, and delivering a female child may be the explanation for the increased odds of DV; however, it is beyond the scope of this paper. This association between having live children and increased odds of DV raises intriguing questions about the gender dynamics within families, warranting further exploration. Husbands with a habit of drinking were twice as likely to commit DV at a p value of less than 0.0001. Various other studies described similar findings [8,14,26-29].

Community-level factors, such as caste, religion, and place of residence, also emerge as significant determinants of DV. Women in rural India were more likely to experience DV with odds of 1.13; this finding, though, is in line with a systematic review [26] and NFHS-4 [8] but in contrast to NFHS-3, which reported urban residence as a risk factor [14]. Muslim women were 2.51 times more likely to suffer from DV in comparison to non-Hindu women. A similar picture was also portrayed by the findings of NFHS-3 [14] and NFHS-4 [8,13]. Concerning caste, DV was least likely to occur among ST women with an AOR of 0.82; a similarly lower risk for ST women was reported by Garg et al. [30] in Delhi. Contrasting findings were reported by Singh et al. in Uttar Pradesh [27]. Schedule caste women have the highest risk, and this finding is similar to those of NFHS-3 and 4 [8,14,17]. The rural-urban divide, religious affiliation, and caste dynamics all contribute to the issue's complexity. Muslim women and those from SCs face heightened risks, emphasizing the importance of intersectionality in understanding DV within diverse social frameworks.

Concisely, this study provides a comprehensive exploration of DV in India, considering a myriad of factors that underscore the importance of diversity. It reinforces the need for targeted interventions that acknowledge regional, educational, occupational, and societal nuances to address and prevent DV effectively.

Strength and limitation

In comparison to other surveys, the NFHS-5 offers some important benefits. For starters, it is nationally representative, which allows results to be extended to the entire country. Second, the sample process and instruments utilized correspond to institutional review board-approved ethical guidelines and scientific standards for DV research. Data collectors were trained extensively to observe interview norms based on WHO recommendations for DV data collecting safety precautions. The major basis for discrimination in India has been documented to be caste, with STs and castes facing marginalization. NFHS-5 has considered this, and while choosing clusters, the proportion of SC/ST families was considered, giving them representativeness.

However, the NFHS-5 DV module has limitations. Demographic and Health Surveys still underestimate the extent of intimate partner violence when compared with other surveys, such as the WHO’s multicounty survey on GBV and other specialized violence surveys [14]. In addition, the fact that women in rural India might not be open to discussion on such sensitive issues could be a factor. As a result, the frequencies listed here may be understated.

## Conclusions

Even though India has seen rapid development in recent decades, climbing to be among the top five economies in the world, the menace of DV still looms large in the country. The prevalence estimates are similar to that of NFHS-4 conducted in 2015-2016, underlying the lack of progress in eliminating this grave social pathology. Simple interventions like higher education are high-yielding, but states have failed to improve the education scenario, particularly in the lower socioeconomic classes. As more and more females look for employment not only for independence but also to contribute financially to the family, the patriarchal structure of Indian families feels threatened. Banning alcohol seems impractical and highly unlikely. In this context, education on safe drinking practices might yield positive results. The goal of eliminating violence against women seems a far fetch at this moment and significant targeted efforts are needed to achieve the sustainable development goals target 5.2.1.
